# Mining kidney toxicogenomic data by using gene co-expression modules

**DOI:** 10.1186/s12864-016-3143-y

**Published:** 2016-10-10

**Authors:** Mohamed Diwan M. AbdulHameed, Danielle L. Ippolito, Jonathan D. Stallings, Anders Wallqvist

**Affiliations:** 1 Department of Defense Biotechnology High Performance Computing Software Applications Institute, Telemedicine and Advanced Technology Research Center, U.S. Army Medical Research and Materiel Command, 504 Scott Street, Fort Detrick, MD 21702 USA; 2 U.S. Army Center for Environmental Health Research, 568 Doughten Drive, Fort Detrick, MD 21702 USA

**Keywords:** Acute kidney injury, Toxicogenomics, Kidney co-expression modules, Gene signature, *Havcr1*, KIM-1, Frequently co-expressed genes, AKI pathways, Immunoproteasome, *Cd44* ectodomain, AKI networks

## Abstract

**Background:**

Acute kidney injury (AKI) caused by drug and toxicant ingestion is a serious clinical condition associated with high mortality rates. We currently lack detailed knowledge of the underlying molecular mechanisms and biological networks associated with AKI. In this study, we carried out gene co-expression analyses using DrugMatrix—a large toxicogenomics database with gene expression data from rats exposed to diverse chemicals—and identified gene modules associated with kidney injury to probe the molecular-level details of this disease.

**Results:**

We generated a comprehensive set of gene co-expression modules by using the Iterative Signature Algorithm and found distinct clusters of modules that shared genes and were associated with similar chemical exposure conditions. We identified two module clusters that showed specificity for kidney injury in that they *1*) were activated by chemical exposures causing kidney injury, *2*) were not activated by other chemical exposures, and *3*) contained known AKI-relevant genes such as *Havcr1*, *Clu*, and *Tff3*. We used the genes in these AKI-relevant module clusters to develop a signature of 30 genes that could assess the potential of a chemical to cause kidney injury well before injury actually occurs. We integrated AKI-relevant module cluster genes with protein-protein interaction networks and identified the involvement of immunoproteasomes in AKI. To identify biological networks and processes linked to *Havcr1*, we determined genes within the modules that frequently co-express with *Havcr1*, including *Cd44*, *Plk2*, *Mdm2*, *Hnmt*, *Macrod1*, and *Gtpbp4*. We verified this procedure by showing that randomized data did not identify *Havcr1* co-expression genes and that excluding up to 10 % of the data caused only minimal degradation of the gene set. Finally*,* by using an external dataset from a rat kidney ischemic study, we showed that the frequently co-expressed genes of *Havcr1* behaved similarly in a model of non-chemically induced kidney injury.

**Conclusions:**

Our study demonstrated that co-expression modules and co-expressed genes contain rich information for generating novel biomarker hypotheses and constructing mechanism-based molecular networks associated with kidney injury.

**Electronic supplementary material:**

The online version of this article (doi:10.1186/s12864-016-3143-y) contains supplementary material, which is available to authorized users.

## Background

Acute kidney injury (AKI) is a clinically relevant disorder associated with high rates of morbidity and mortality [1]. It reportedly occurs in ~20 % of hospitalized patients and in 30–60 % of critically ill intensive care patients [2, 3], increases mortality in military-relevant burn causalities [4], and often progresses to chronic kidney disease [3]. In the United States, the annual costs for hospital-acquired AKI are estimated to be greater than $10 billion [5], and recent epidemiological studies show a trend towards increasing occurrence of AKI [6–8]. The lack of suitable biomarkers is a major hurdle in timely diagnosis of AKI, especially because drug-induced AKI is often reversible as long as drug use is discontinued.

Functional markers, such as serum creatinine, blood-urea-nitrogen, and the volume of urine output, are currently used to diagnose AKI [2, 9]. These markers have low sensitivity, lack specificity, result in delayed diagnosis, and hence, contribute to poor clinical outcomes [2, 10]. Developing suitable pre-clinical markers that could aid in earlier identification of AKI will also help reduce the cost and time associated with advanced drug development [11]. The Predictive Safety Testing Consortium recently addressed this issue and developed the first U.S. Food and Drug Administration (FDA)-approved AKI biomarker panel for use in pre-clinical studies [12]. While this is a major advance in the field, our current knowledge of molecular mechanisms and networks associated with AKI remains incomplete [13], and the search for new clinical AKI biomarkers is ongoing [9, 14]. Thus, identifying new molecular-level insights of kidney injury will help us to not only understand the disease process but also to identify new candidate biomarkers.

Extraction of network and pathway information by using in vivo gene expression datasets from thousands of chemical exposures and disease conditions can provide detailed insight into molecular injury mechanisms and identify the corresponding mechanism-based biomarkers [15–20]. The diversity and complexity of the in vivo response require specialized techniques to extract interpretable biological information. Here, we used the Iterative Signature Algorithm (ISA) to generate gene co-expression modules associated with AKI [21, 22]. Co-expressed genes are hypothesized to participate in biological processes and pathways that are linked together, though not necessarily through gene co-regulation. The bi-clustering methodology identifies gene modules that are clustered together under a subset of conditions, such that modules that can be specifically associated with kidney disease conditions can be hypothesized to be linked to kidney injury mechanisms. In this formulation, individual genes can participate in more than one module, because the same gene will respond differently to different stimuli, leading to co-expression with different sets of genes [23]. This is consistent with the concept of a molecular toxicity pathway, in which a limited number of pathways are differentially activated in response to different injury conditions.

We investigated the DrugMatrix toxicogenomics database, which contains chemically induced gene expression changes and associated clinical chemistry and organ-specific histopathology endpoints in male Sprague Dawley rats [24, 25]. The DrugMatrix kidney Affymetrix dataset has ~60 million in vivo gene expression data points associated with diverse chemical exposures. We have previously used DrugMatrix liver data to identify modules and networks associated with liver fibrosis and experimentally validated the identified liver fibrosis gene signature [26–28]. Fielden et al. utilized part of the DrugMatrix kidney data to develop a predictive gene signature for kidney injury [29], but to our best knowledge, there are no reports on identifying co-expression modules associated with kidney injury from toxicogenomic data. Thus, our goal in this study was to identify co-expression modules associated with AKI. Our study was based on two major hypotheses: *1*) a bi-clustering approach should be able to identify toxicity-relevant co-expression modules in an unsupervised manner, and *2*) genes consistently co-expressed with known biomarker candidates should identify biological networks associated with kidney injuries. We identified AKI-relevant modules that were activated in chemical exposure conditions causing kidney injury and which contained well-known AKI-relevant genes such as *Havcr1*, *Clu*, and *Tff3*. We used the genes in these modules to *1*) generate a signature for predicting kidney injury that performed better than well-known AKI biomarkers, and *2*) identify pathways and networks of extracellular matrix (ECM)-receptor interactions, glutathione metabolism, and p53 signaling pathways associated with kidney injury. Our network analysis identified the involvement of immunoproteasomes in AKI. To expand on the knowledge and pathways associated with the well-known AKI gene *Havcr1* [also known as the kidney injury molecule-1 (KIM1)], we identified genes that were frequently co-expressed with *Havcr1* under conditions that cause kidney injury. We also confirmed that the expression pattern of these genes was present in an independent dataset probing rat kidney injuries due to ischemia, not chemical exposures. These results illustrate the potential of our approach to identify molecular networks associated with toxic injury and as a potential source for biomarkers.

## Methods

### Data collection and preprocessing

We used data from DrugMatrix, a publicly available toxicogenomics database that contains matched gene expression and histopathology data from Sprague Dawley rats after exposure to a range of chemicals at different doses and time intervals [24]. We downloaded the DrugMatrix data from the National Institute of Environmental Health Sciences [30] server and focused on the kidney data generated using the Affymetrix rat 230 2.0 GeneChip array. We followed the pre-processing protocol as described in our earlier study [26, 31]. Briefly, we used the R/BioConductor package *affy* to perform robust multi-array average quantile normalization and the BioConductor package *ArrayQualityMetrics* to assess the quality of the microarray data [32–34]. We renormalized the data after removing 97 arrays that failed on at least one of the three statistical tests in *ArrayQualityMetrics* and were thus identified as outliers. We used the *MAS5calls* function in the *affy* package to obtain the “Present/Absent” calls for each probe set and removed the probe sets that were found to be “Absent” in all replicates across all chemical exposures [31].

We used the BioConductor *genefilter* package to remove genes without EntrezIDs or with low variance across chemical exposures [35]. After calculating the average intensity across the replicates of a chemical exposure condition, we computed log-ratios for each gene between treatments and their corresponding controls. In the current analysis, we focused on chemical exposures with greater than 1-day time-points. This resulted in a final log-ratio matrix of 9,222 genes and 220 chemical exposure conditions.

### Generation of gene co-expression modules and module clusters

We used the ISA implemented in the R/BioConductor package *eisa* to generate gene co-expression modules [36]. The three key parameters of this algorithm are *1*) starter seeds, *2*) gene threshold, and *3*) condition threshold. We used the R hierarchical clustering package *Hclust* along with the *dynamicTreeCut* package and generated 216 gene clusters [37]. In line with our previous work, random gene sets were added to the hierarchical gene clusters and expanded to ~15,000 gene sets [28]. We used both the hierarchical gene clusters and expanded gene sets as the starter seeds.

The ISA uses gene and condition threshold parameters for module generation. These two parameters affect the size and stringency of the modules; i.e., the higher their values, the smaller and more highly correlated are the modules, whereas the smaller their values, the larger and less-correlated the modules [23]. We varied the parameter combination from 2.0 to 4.0 in increments of 0.5, and used 25 combinations of these two parameters to generate the modules; i.e., for a gene threshold of 2.0, we analyzed the modules at condition thresholds of 2.0, 2.5, 3.0, 3.5, and 4.0. For each threshold combination, we normalized the log-ratio matrix, generated modules, filtered redundant modules, and ensured module robustness by using the *ISAnormalize*, *ISAiterate*, *ISAunique*, and *ISAFilterRobust* functions, respectively. We filtered out gene modules with more than 200 genes and an intra-module correlation of <0.4. Finally, we merged the modules generated by using all threshold combinations and removed any redundant modules with the *ISAunique* function. Using this procedure, we generated 137 gene co-expression modules. Additional file 1: Script S1 provides the R script used to generate the ISA modules.

Overlap of genes and chemical exposures is permitted in the co-expression modules generated using the process above. Quantifying the level of overlap allowed us to group similar modules together. Toward this end, we calculated the overlap of gene and chemical exposures in modules, using the Dice coefficient [38, 39]. Equation 1 shows how we calculated the module overlap score (OS_A,B_) between two modules A and B.

1 $$ {\mathrm{OS}}_{\mathrm{A},\mathrm{B}}=\left(2*\frac{\mid \mathrm{G}\left(\mathrm{A}\right)\ \cap\ \mathrm{G}\left(\mathrm{B}\right)\mid }{\mid \mathrm{G}\left(\mathrm{A}\right) \mid + \mid \mathrm{G}\left(\mathrm{B}\right)\mid}\right)+\left(2*\frac{\mid \mathrm{E}\left(\mathrm{A}\right)\ \cap\ \mathrm{E}\left(\mathrm{B}\right)\mid }{\mid \mathrm{E}\left(\mathrm{A}\right) \mid + \mid \mathrm{E}\left(\mathrm{B}\right)\mid}\right) $$

Here, |G(A)| represents the count of genes in module A; |G(B)| that of genes in module B; |E(A)| that of chemical exposures in module A; |E(B)| that of chemical exposures in module B; |G(A)∩G(B)| that of genes in common between modules A and B; and |E(A)∩E(B)| that of chemical exposures in common between modules A and B. The overlap score varies between zero and two, where zero represents no overlap and two represents complete overlap between the two modules. We created a module overlap score matrix by calculating the module overlap scores between all 137 co-expression modules and performing hierarchical clustering by using the *Hclust* package in R. The generated dendrograms were cut at a specific height (h = 0.5), which resulted in 16 final module clusters.

### Module cluster characterization

We used activation scores and enrichment of known AKI-relevant genes to identify AKI-relevant module clusters. We followed the procedure described in our earlier work to calculate the module cluster activation scores [26]. Briefly, we first normalized the values in the log-ratio matrix of each gene across 220 chemical exposures by converting them to Z-scores. The Z-score of gene *i* under chemical exposure condition *j* is given by

2 $$ {\mathrm{Z}}_{i,j} = \frac{X_{i,j} - \kern0.75em {\mu}_i}{\sigma_i}, $$

Where *X*_*i,j*_ is the log-ratio value for gene *i* under chemical exposure condition *j*; *μ*_*i*_ is the average log ratio for gene *i* across all 220 chemical exposures; and *σ*_*i*_ is the standard deviation of the log ratio for gene *i* across all 220 chemical exposures. Next, we defined eight phenotypes, two of which were histopathological phenotypes based on available kidney histopathological data, and the remaining six of which were chemical exposure classes based on the pharmacological/toxicological class of the chemicals. The histopathological phenotypes were *kidney-cortex, tubule, necrosis* (P1), and *kidney-tubule regeneration* (P2). In these two cases, we chose a chemical exposure to be representative of the phenotype if all of its replicates had a histopathology score of ≥2 for the given phenotype.

We defined six chemical exposure classes (hepatotoxicants, fluoroquinolone antibiotics, epidermal growth factor receptor kinase inhibitors, estrogen modulators, statins, and fibrates) based on the pharmacological class, and chose the chemical exposures with the highest dose for each chemical within a chemical exposure class as representatives. We provide the chemical exposures used to define each phenotype/chemical exposure class in Additional file 2: Table S1. The activation score *A*_*m*,*p*_ of module cluster *m* associated with phenotype *p* is the mean absolute value of the Z-score for all genes *i* in module cluster *m* across all conditions *j* in phenotype *p* and is given by

3 $$ {A}_{m,p}=\frac{1}{N_m{N}_p}{\displaystyle {\sum}_{i\in m}^{N_m}{\displaystyle {\sum}_j^{Np}\left|{Z}_{i,j}\right|}}, $$

Where *N*_*m*_ is the number of genes associated with module cluster *m*; *N*_*p*_ is the number of chemical exposures associated with phenotype *p*; and Z_*i,j*_ is the Z-score of gene *i* under chemical exposure condition *j* associated with phenotype *p*.

We collected 57 genes with direct evidence of association with AKI from the Comparative Toxicogenomics Database (CTD) [40], 25 of which mapped to the co-expression modules. We used Fisher’s exact test to calculate the enrichment of these genes in each module cluster. In this process, we identified two module clusters (MC7 and MC11) that were activated in kidney injury conditions and enriched with known AKI-relevant genes. We refer to these two module clusters as the AKI-relevant module clusters and their component genes as the AKI-relevant gene set.

### Generation of gene signature to predict kidney injury

We utilized the AKI-relevant gene set and generated gene signatures to predict the future occurrence of kidney injury, using the R package *randomForest* [41]. Fielden et al. reported chemical exposures that did not show histopathological kidney injury at 5-day exposure but did show kidney injury at 29-day exposure [29]. Our training set, based on these definitions, consisted of 14 nephrotoxic chemical exposures at early time points and 30 non-nephrotoxic chemical exposures [29]. We used the AKI-relevant gene set and developed a random forest-based classifier model, using 1,001 trees and default settings. To analyze model performance, we used the standard model evaluation parameters, such as sensitivity, specificity, and area under the curve (AUC) for the receiver-operator characteristic (ROC) curve. The *ROCR* package was used to generate the AUC-ROC curve [42]. We generated the final model by using the top 30 genes identified in the initial run. We separately created models with the *Havcr1* and *Clu* genes, using the same parameters as those above. We utilized an independent external dataset from the Toxicogenomics Project-Genomics Assisted Toxicity Evaluation System [TG-GATEs] to evaluate the 30-gene signature [43]. We processed 4- and 8-day high dose kidney exposures from TG-GATEs, using the same procedure as that described above for processing the DrugMatrix dataset.

### Functional enrichment analysis

We utilized the AKI-relevant gene set and carried out functional enrichment analyses of Kyoto Encyclopedia of Genes and Genomes (KEGG)/Reactome pathways and Gene Ontology-Biological Process (GO-BP) terms. We used the BioConductor package *clusterProfiler* with default settings for KEGG and GO-BP enrichment analysis [44], and the REVIGO webserver with the default semantic similarity measure (SimRel) to cluster and summarize the enriched GO-BP terms [45]. We employed the *ReactomePA* package for Reactome pathway enrichment analysis [46].

### Protein-protein interaction network analysis

We converted the rat Affymetrix probe IDs to human gene IDs using the BioConductor/R packages *annotate* and *biomaRt* [47]. The R script used for the conversion of probe IDs to human gene IDs is provided in additional information (Additional file 3: Script S2). We used the high-confidence human protein-protein interaction (PPI) network with 14,862 unique nodes generated by using interaction detection based on the shuffling approach [48]. We used Cytoscape 3.2.1 to map the AKI-relevant gene set to the PPI network, and extracted the connected component as an AKI-relevant sub-network (AKI-SN) [49]. We first analyzed whether the AKI-SN was obtained by random chance using by two statistical significance tests [26, 50]. Our null hypothesis was that the observed number of nodes (AKI-SN_nodes_) and edges (AKI-SN_edges_) in AKI-SN are obtained by random chance. In the first analysis, we randomly selected 158 proteins from the human PPI network 1,000 times and counted the number of nodes (R_nodes_) and edges (R_edges_) of the largest connected component. We calculated the number of times that R_nodes_ ≥ AKI-SN_nodes_ (N_randnode_). Similarly, we computed the number of times that R_edges_ ≥ AKI-SN_edges_ (N_randedge_). We then calculated the probability of randomly obtaining a sub-network with a number of nodes comparable to that of AKI-SN by P = N_randnode_ /1,000, and the probability of randomly obtaining a sub-network with a number of edges comparable to that of AKI-SN by P = N_randedges_ /1,000. In the second analysis, we scrambled the human PPI network while preserving the average node degree and mapped AKI-SN nodes to the randomized network. This process was also repeated 1,000 times. As in the first test, we extracted the largest connected component, counted the number of nodes and edges, and calculated the probability of obtaining AKI-SN parameters by random chance.

We next computed the properties of the network, such as its *degree* and *betweenness centrality*. In the PPI network, nodes represent proteins and edges represent the interactions/connections between them. The *degree* represents the number of interactions associated with the protein. Proteins with a large *degree* are known as hub proteins [51]. Earlier studies have shown that hub proteins tend to be the essential or key protein in the network [52]. The *betweenness centrality* (also known as *traffic*) is a measure of the number of shortest paths through the node and represents the capacity of the node to communicate with other components of the network [51]. We used the *NetworkAnalyzer* option in Cytoscape 3.2.1 to compute the *degree* and *betweenness centrality* of the AKI-SN [53]. We used the *MCODE* program in Cytoscape to identify the most inter-connected nodes in the AKI-SN [54].

The Ingenuity Pathways Analysis (IPA; QiagenBioinformatics, Redwood City, CA) software package was used to determine the gene-function relationships and sub-anatomical location of the genes and proteins identified in the sub-networks. The genes identified in the sub-network analysis were associated with the disease or function annotations filtered by the search term “renal” in order to determine the gene-to-function relationships in discrete anatomical locations of the kidney. Only PubMed identifications (PMIDs) with significant associations curated by IPA were included in the anatomical mapping.

### Identification of frequently co-expressed genes

Of the 137 gene co-expression modules, 21 contained the *Havcr1* gene. We created a correlation matrix for each of the 21 modules and selected the 20 % of genes that were most correlated with *Havcr1*. We counted the number of occurrences of each gene across the list of the 21 most correlated gene sets, and genes that occurred more than twice were denoted as “frequently co-expressed genes.” We tested whether these frequently co-expressed genes could be obtained by random chance. For this, we shuffled the gene labels in the log-ratio matrix and carried out the entire analysis from the ISA module generation to identify frequently co-expressed genes. Subsequently, we analyzed whether the procedure was robust with respect to noise. We repeated the entire analysis excluding either 5 % of the chemical exposures or 10 % of chemical exposures and calculated the genes frequently co-expressed with *Havcr1*.

### External validation

We further evaluated the relevance of frequently co-expressed genes with *Havcr1*, using an external dataset (GSE58438) collected from Gene Expression Omnibus (GEO). In this dataset, ischemic kidney injury was produced by clamping the renal artery [55]. This dataset contains five control replicates and five replicates of animals collected at 1 and 5 days after ischemic kidney injury. We matched the frequently co-expressed genes and calculated the Spearman correlation between the average log-ratios derived from chemical exposures that produced kidney injury in the DrugMatrix database and those derived from this external dataset.

## Results and discussion

### Identification of AKI-relevant modules

**Fig. 1:**
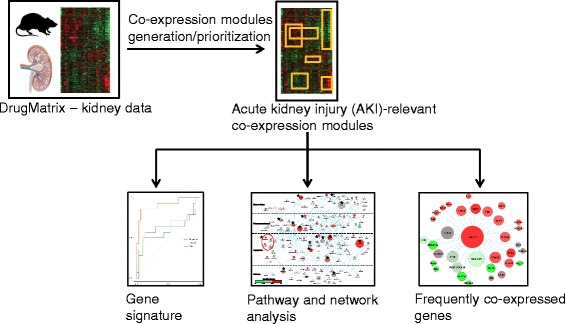
Workflow used in this study to mine kidney toxicogenomic data

**Fig. 2:**
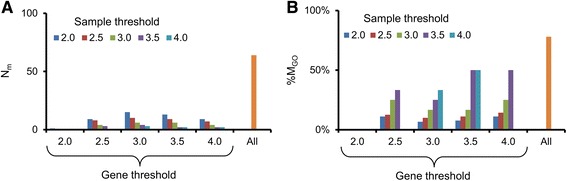
Iterative signature algorithm (ISA) parameter selection. **a** Number of modules generated for different combinations of gene and sample thresholds and for the merged results. **b** Percentage of modules enriched with gene ontology (GO) terms for different threshold values and merged results. “All,” merged result from all threshold combinations; %M_GO_, the percentage of modules enriched with GO terms; N_m_, the number of modules generated

Figure 1 shows the overall workflow used in this study. We pre-processed DrugMatrix kidney toxicogenomic data and obtained a final matrix of 9,222 genes arrayed across 220 chemical exposure conditions. We constructed co-expression modules by using the ISA approach and systematically varying the parameters of gene and condition thresholds from 2.0 to 4.0 in increments of 0.5 (*n* = 25 different threshold combinations; Fig. 2). We also performed a separate analysis merging all modules generated from each threshold combination, removing identical or redundant modules. The merged set resulted in the highest percentage of modules enriched with at least one GO term for the bi-clustering analysis (Fig. 2b). Based on this analysis, we used the merged set containing 137 gene co-expression modules in our study (see Additional file 4: Table S2 and Additional file 5: Table S3 for a detailed list of the genes and chemical exposures, respectively, associated with each of the 137 modules). This approach of using the combination of all threshold parameters affords the advantages of being unsupervised and non-parametric, and overcomes potential biases inherent in using a single parameter value.

**Fig. 3:**
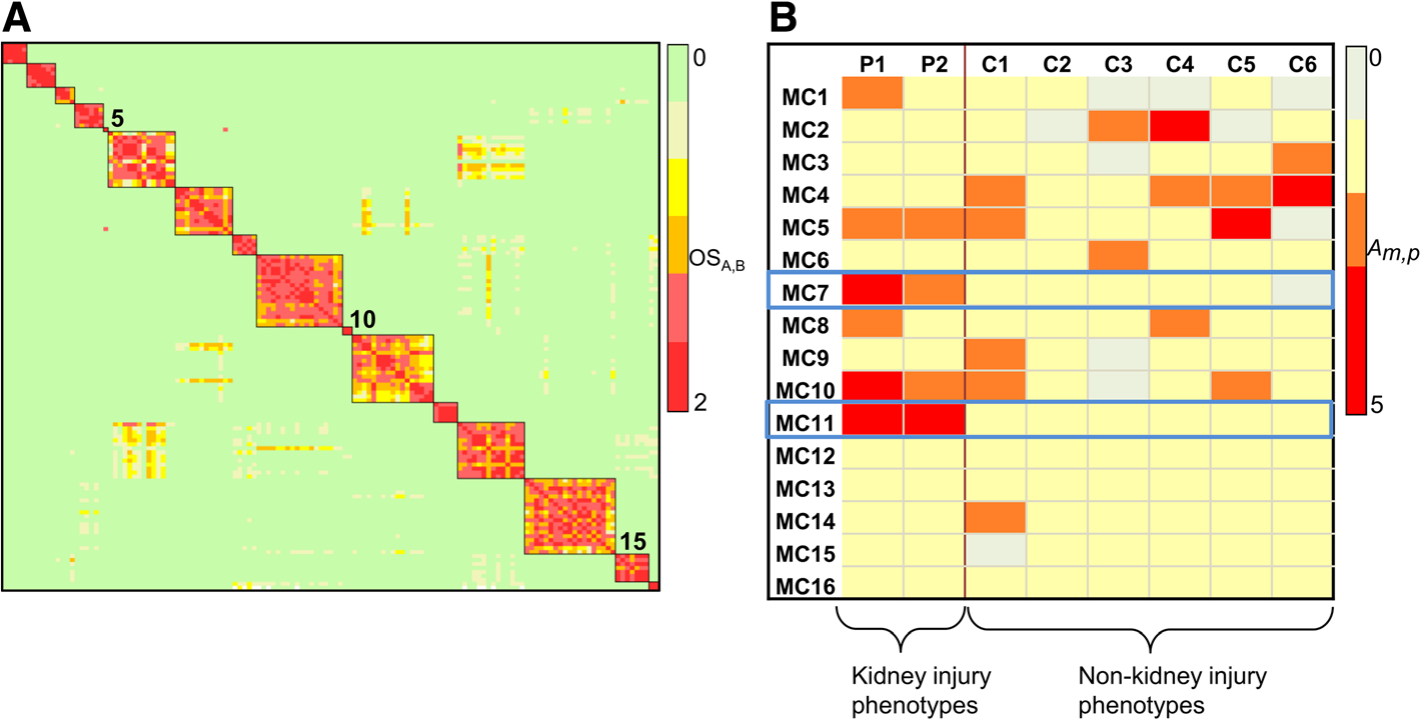
**a** Heat map view of modules clustered based on the module overlap score. **b** Activation of module clusters (MC1-16) for different phenotypes (P1-2) and chemical classes (C1-6). P1, chemical exposures that cause kidney-cortex, tubule, necrosis; P2, chemical exposures that cause kidney-tubule, regeneration; C1, chemical exposure known to cause hepatotoxicity; C2, fluoroquinolone antibiotics; C3, epithelial growth factor receptor kinase inhibitors; C4, estrogen receptor modulators; C5, high dose of statin drugs; C6, high dose of anti-lipidemic drugs (fibrates)

The co-expression modules differ from conventional clusters by allowing the same genes and chemical exposures to be present in multiple modules. We quantified this overlap between module genes and chemical exposures by using the Dice coefficient (Eq. 1). We clustered the resultant module overlap score matrix, allowing us to condense the original 137 modules into 16 distinct module clusters with high intra-module correlations and low inter-module correlations (Fig. 3a). We identified AKI-relevant modules by evaluating the module cluster activation across two different kidney histopathologies (P1-P2) and six pharmacological classes of drug or toxicant exposure that did not produce kidney pathology (C1-C6) (Table 1). Module clusters 7 and 11 showed specificity for kidney injury, being activated in kidney injury phenotypes (i.e., P1 [kidney cortical necrosis] or P2 [kidney tubular regeneration]) but not after exposure to drugs or toxicants unassociated with kidney injury (i.e., C1-C6). Module cluster 10 was excluded from further analysis because although it was activated in kidney injury phenotypes P1-P2, it was also non-specifically activated by compounds not associated with kidney injury (i.e., hepatotoxicants [C1] and statins [C4]). We further analyzed module clusters 7 and 11 to verify that they contained genes recognized in the literature to be associated with AKI. We calculated the enrichment of the AKI-associated genes identified in CTD and mapped them to the module clusters (Table 2). AKI-relevant genes, including *Havcr1*, *Clu*, and *Tff3*, mapped to module clusters 7 and 11 but not to other clusters. Thus, the AKI-relevant gene sets that comprise these modules are activated in conditions producing kidney pathology, specific to kidney injury, and enriched in genes associated with AKI (see Additional file 6: Table S4 for the 679 AKI-relevant genes along with their log-ratio values in the three chemical exposures that produced the kidney necrosis phenotype (P1)). Taken together, these data suggest that both modules are relevant to AKI.

**Table 1:** Phenotypes and chemical exposure classes used in the analysis of module cluster activation

Name	Class
P1^a^	Kidney-cortex, tubule, necrosis
P2^a^	Kidney-tubule, regeneration
C1	Hepatotoxicants
C2	Fluoroquinolone antibiotics
C3	Epithelial growth factor receptor kinase inhibitors
C4	Estrogen receptor modulators
C5	Statins
C6	Fibrates

^a^P1 and P2 were defined based on histopathology data

**Table 2:** Enrichment of known acute kidney injury (AKI)-relevant genes in module clusters

Module cluster	No. of genes in module cluster	No. of AKI genes	*p*-value	AKI-relevant genes
1	184	0	1.00	-
2	164	1	0.67	*CYP2D6*
3	217	7	0.0008	*ALB, AMBP, CYP2C19, CYP2C9, CYP2D6, FABP1, GSTP1*
4	181	0	1.00	-
5	70	0	1.00	-
6	286	1	0.88	*PPARG*
7	273	7	0.002	*B2M, CD44, GPNMB, G6PD, GSTP1, HAVCR1, TFF3*
8	197	1	0.78	*HEXB*
9	320	0	1.00	-
10	136	0	1.00	-
11	517	10	0.002	*A2M, CLU, CD44, GPNMB, GAS6, HAVCR1, LCN2, SPP1, TNFRSF12A, TFF3*
12	217	0	1.00	-
13	509	2	0.89	*AMN, OCLN*
14	509	5	0.34	*A2M, EPO, GSTM2, LCN2, SPP1*
15	379	0	1.00	-
16	181	0	1.00	-

To our best knowledge, this study is the first module-based analysis of a large public repository of kidney toxicogenomic data. Our unsupervised approach is an advantage in that, unlike standard differential gene expression analysis, co-expression modules are not associated a priori with any injury phenotype. The modules we generated represent a compendium of gene co-expression patterns in rat kidney tissue after exposure to diverse toxicants (Additional file 4: Table S2). We used disease-relevant genes *post hoc* to prioritize modules rather than following previously published methods that use known disease-relevant genes to guide the co-expression analysis a priori [56]. Our module activation calculations and enrichment analyses did not influence the gene/condition composition of the modules because they were performed after we generated the modules. As such, the module genes, i.e., genes co-expressed with known AKI-relevant genes, can be hypothesized to participate in biological processes/disease mechanisms relevant to kidney-specific injuries. When we analyzed the chemical exposures that define the modules in AKI-relevant module clusters, the results included well-known nephrotoxic chemicals/drugs (e.g., cisplatin, lead-II-acetate, calcitriol, cholecalciferol, netilmicin, vancomycin, and oxaliplatin; Additional file 7: Table S5) [5]. We used the AKI-relevant gene set to carry out two separate analyses: *1*) identification of gene signatures by using a supervised classification approach; and *2*) identification of the pathways and networks associated with AKI.

### Generation of predictive gene signature for AKI

We used the AKI-relevant gene set along with the random forest approach to generate gene signatures that could predict the future onset of AKI. Although the nephrotoxicants in our training data did not show any kidney injury at 3 to 5 days of exposure, they are known to produce kidney injury after 28 days of exposure [29]. Thus, the task is to use the data at early time points to predict the probability of injury before the injury actually occurs. We used the random forest approach to predict the probability of later injury on the basis of the data at early time points. The random forest approach is an ensemble-based decision-tree model that has been widely used in the analysis of omics data [57–60]. The main advantages of this approach are its ability to *1*) handle problems with small sample sizes and a large number of variables and *2*) provide a measure of variable importance [57]. In the random forest approach, each tree is generated with a random subset of the data and a prediction is made for the left-out data that are not used in the tree generation. This out-of-bag (OOB) testing provides an error estimate and is equivalent to cross-validation analysis [60]. Our random forest approach identified the top 30 genes that provide the best classification accuracy with the variable importance measure (i.e., the mean decrease in accuracy; see Additional file 8: Table S6).

**Fig. 4:**
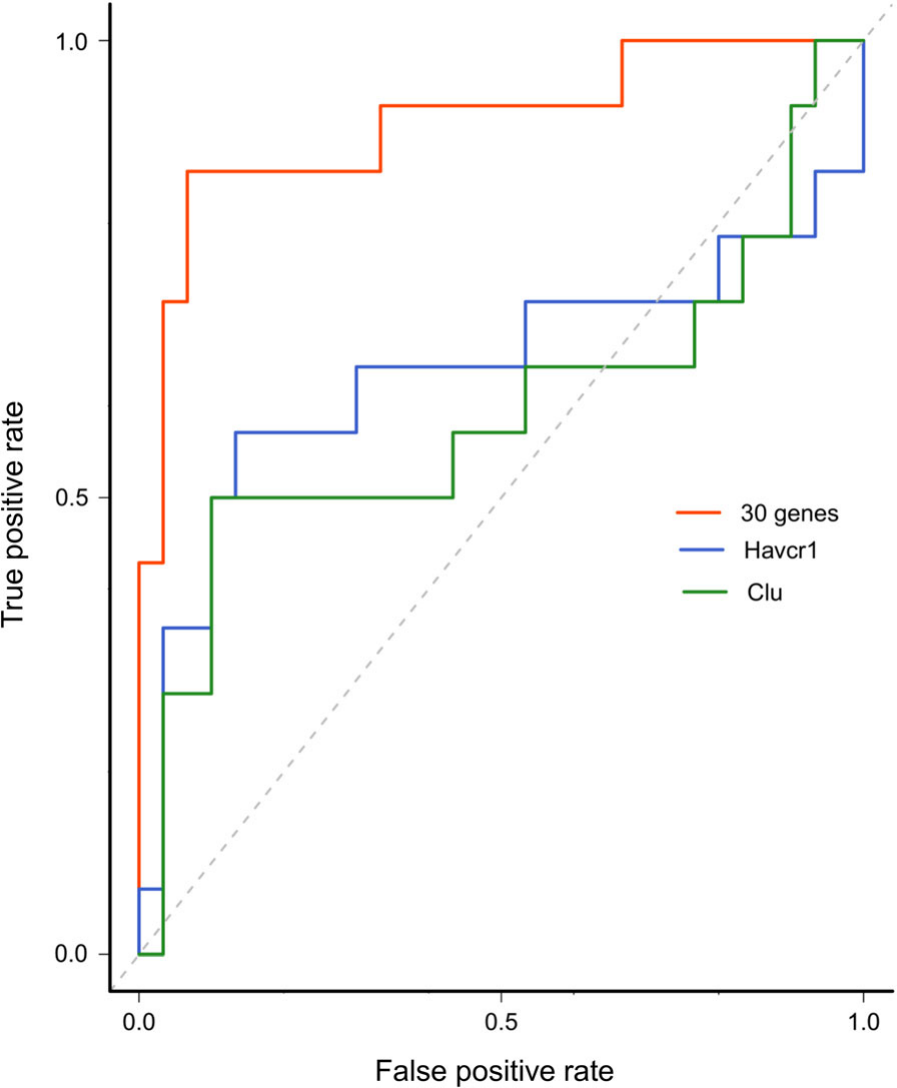
Receiver Operator Characteristics for the 30-gene signature, *Havcr1*, and *Clu*. The model uses early (1–5 days) transcription data to predict the future onset of kidney injury (at 28 days). The true positive rate is the rate of true predictions divided by true predictions and false positives; the false positive rate is the rate of false predictions divided by the false predictions and false negatives. The diagonal line indicates random predictions

Next, we used these 30 genes to develop a classification model. The final model had a sensitivity of 86 %, specificity of 93 %, AUC of 0.91, and OOB error estimate of 9.1 % (Fig. 4). This performance estimate is overly optimistic, because the 30-gene signature was evaluated by using the same set as that used in gene prioritization [43]. We compared the performance of this signature with known biomarkers by developing separate models, using just the *Havcr1* or *Clu* gene. In ROC curve comparisons, the 30-gene signature model had better predictive potential than models that used only the *Havcr1* or *Clu* gene (Fig. 4). Importantly, our gene signature included both genes previously associated with kidney injury (e.g., *Havcr1*) and potentially new biomarkers of kidney injury (e.g., *Irf6*). The model with *Havcr1* alone had a sensitivity of 57 %, specificity of 80 %, AUC of 0.64, and OOB error estimate of 27.3 %. The model with *Clu* alone had a sensitivity of 50 %, specificity of 66.7 %, AUC of 0.59, and OOB error estimate of 38.7 %. The 30-gene signature model had a sensitivity of 83 %, specificity of 75 %, and accuracy of 79 % in classifying an independent external dataset of 38 chemical exposures with 3 days of exposure duration (see Additional file 9: Table S7). Thus, overall the model shows good performance in predicting the future onset of kidney injury. We observed a decrease in performance at later time points after the injury was well-established (see Additional file 9: Table S7). Overall, our results are comparable to those of a prior study reporting that a 35-gene signature predicted kidney injury by using a different array platform. However, 13 of 35 of the genes in that study could not be identified and were labeled only as EST [29].

Besides *Havcr1*, the literature includes reports of associations between the genes in our signature and AKI. *Guca2a*, a gene that codes for guanylate cyclase 2a, was upregulated in gentamicin-induced kidney toxicity [61]. The protein encoded by gene *Ugt2b7* [uridine diphosphate glucuronosyltransferase (UGT)], one of the two most abundantly expressed renal UGTs, is known to play a significant role in glucuronidation of drugs and endogenous mediators associated with inflammation [62]. The gene *Ly96* (lymphocyte antigen 96) encodes a protein that interacts with toll-like receptor 4 (TLR4), a key mediator in nephrotoxicity. *Tlr4 -/-* mice are less prone to ischemic kidney injury than are wild-type littermates [63, 64]. TLRs induce the expression of PVR (also known as CD155), another gene present in our gene signature [65]. TLR4 signaling can lead to activation of interferon regulatory factors (IRFs) [65]. *IRF1* promotes inflammation after ischemic AKI [66]. Furthermore, the *Map4k4* gene in our signature encodes a kinase involved in the TNF-signaling pathway, which is also known to play a role in kidney injury [67]. *Irf6* in our signature is functionally related to TLR4 signaling but has not previously been associated with AKI; it may represent a novel biomarker of kidney injury. Thus, the literature provides supporting evidence that all of the genes in our signature are relevant to predicting kidney injury.

### Functional enrichment analysis

We identified the pathways associated with the AKI-relevant gene set, using KEGG and Reactome pathway enrichment analysis (Tables 3 and 4, respectively). The enriched KEGG pathways of ECM-receptor interactions [68, 69], cell adhesion, and focal adhesion pathways represent known disease processes of AKI associated with loss of attachment to the basement membrane, changes in actin cytoskeletal structure, and loss of cell-cell contacts due to redistribution of cell adhesion molecules and integrins [70]. The *p53* signaling pathway plays a key role in cisplatin-induced kidney damage [71]. The bile secretion pathway mapped many transporters associated with kidney injury (e.g., *Slc21a4* and *Abcc2* [72]). Glutathione depletion is consistent with kidney injury [73], and the retinoic acid-inducible gene-1 (*RIG-1*) signaling pathway modulates immune and inflammatory responses in renal diseases [74].

**Table 3:** KEGG pathway enrichment for the acute kidney injury (AKI)-relevant gene set

Pathway	Count	*p*-value^a^
ECM^b^-receptor interaction	14	9.10^−5^
Glutathione metabolism	11	4.10^−4^
Cell adhesion molecules	16	0.012
Bile secretion	10	0.012
Cytosolic DNA-sensing pathway	7	0.026
Cysteine and methionine metabolism	6	0.026
p53 signaling pathway	9	0.026
RIG-I^c^-like receptor signaling pathway	8	0.029
Toxoplasmosis	12	0.039
Amoebiasis	10	0.039
Focal adhesion	15	0.046
Metabolism of xenobiotics by cytochrome P450	8	0.046

^a^ *p*-value after Benjamini-Hochberg multi-test correction, ^b^Extracellular matrix

^c^Retinoic acid-inducible gene-1

**Table 4:** Reactome pathway enrichment for the acute kidney injury (AKI)-relevant gene set

Pathway	Count	*p*-value^a^
Extracellular matrix organization	21	0.001
Degradation of the extracellular matrix	11	0.014
Cell junction organization	9	0.025

^a^ *p*-value after Benjamini-Hochberg multi-test correction

**Fig. 5:**
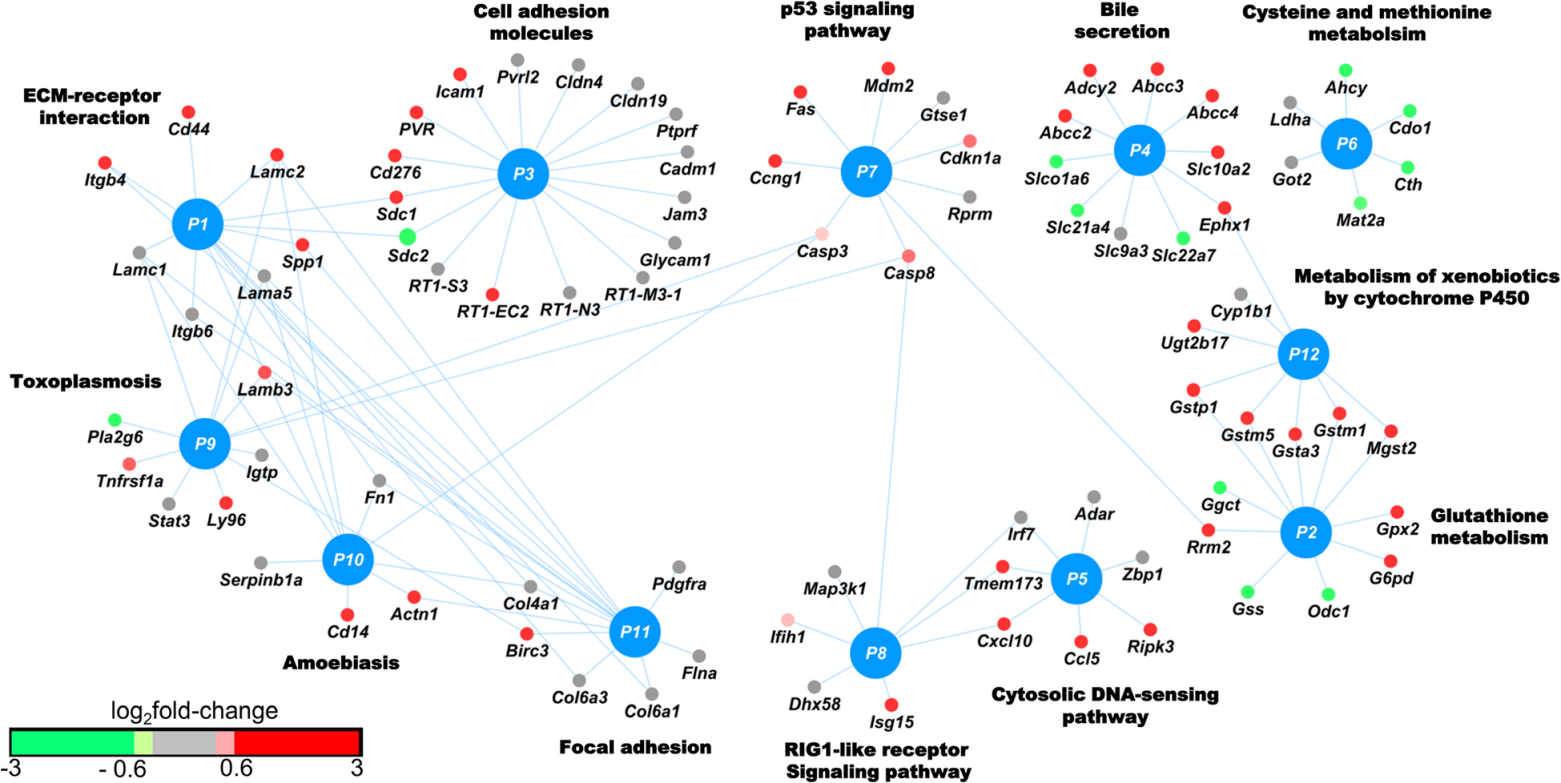
Genes in the acute kidney injury (AKI)-relevant gene set mapped to the enriched Kyoto Encyclopedia of Genes and Genomes (KEGG) pathways. The color of the gene nodes indicates the log_2_-fold change and the connecting lines represent membership in the KEGG pathways (P1-P12) listed in Table 3

We mapped the individual genes in the AKI-relevant gene set to the KEGG pathways listed in Table 3 (Fig. 5). Individual genes reported in AKI are evident in this network (e.g., *Spp1*, *Cd44*, *Gstp1*, *Gstm1*, *Icam1*, *Mdm2, Abcc2*, and *Ccl5* [75–78]). Of note, several genes associated with programmed cell death in AKI [5] mapped to related pathways: the apoptosis-related genes *Fas, Casp3*, and *Casp8* mapped to the *p53* signaling pathway; *Ripk3*, a key mediator of necroptosis, mapped to the cytosolic DNA-sensing pathway; and *Tnfrsf1a*, a gene associated with inflammation and apoptosis, mapped to the toxoplasmosis pathway. Similarly, the enriched GO-BP terms for the AKI-relevant gene set identified AKI-related biological processes, such as the immune response, glutathione metabolism, and cell killing (Table 5). These results suggest that co-expression module-based analysis offers richer information than conventional differential gene expression analysis. Pathway enrichment analysis of the differentially expressed genes in chemical exposures that produce kidney injury (P1 and P2) could only identify a limited number of pathways and missed pathways related to ECM receptor interaction, cell adhesion, the RIG-1-like receptor signaling pathway, and toxoplasmosis (see Additional file 10: Table S8). Moreover, the number of genes that mapped to AKI-relevant pathways was higher in co-expression module-based analysis than in differential gene expression analysis.

**Table 5:** Gene Ontology (GO)-biological process (BP) term enrichment for acute kidney injury (AKI)-relevant gene set^a^

Pathway	log_10_ *p*-value
Immune response	−5.01
Defense response	−4.47
Response to virus	−4.02
Immune system process	−3.57
Response to external stimulus	−3.57
Response to stimulus	−3.57
Response to biotic stimulus	−3.50
Multi-organism process	−3.35
Response to cytokine	−3.32
Regulation of viral process	−3.17
Response to interferon-beta	−2.89
Response to organic substance	−2.79
Viral genome replication	−2.74
Single-organism process	−2.74
Response to chemical	−2.67
Regulation of symbiosis	−2.48
Cellular response to interferon-beta	−2.41
Response to interferon-alpha	−2.39
Glutathione metabolic process	−2.89
Nucleobase-containing small molecule metabolic process	−2.08
Positive regulation of cell-killing	−2.43
Cell-killing	−2.12

^a^Enriched GO-BP terms were clustered and summarized by using REVIGO webserver [45]

### PPI network analysis

**Fig. 6:**
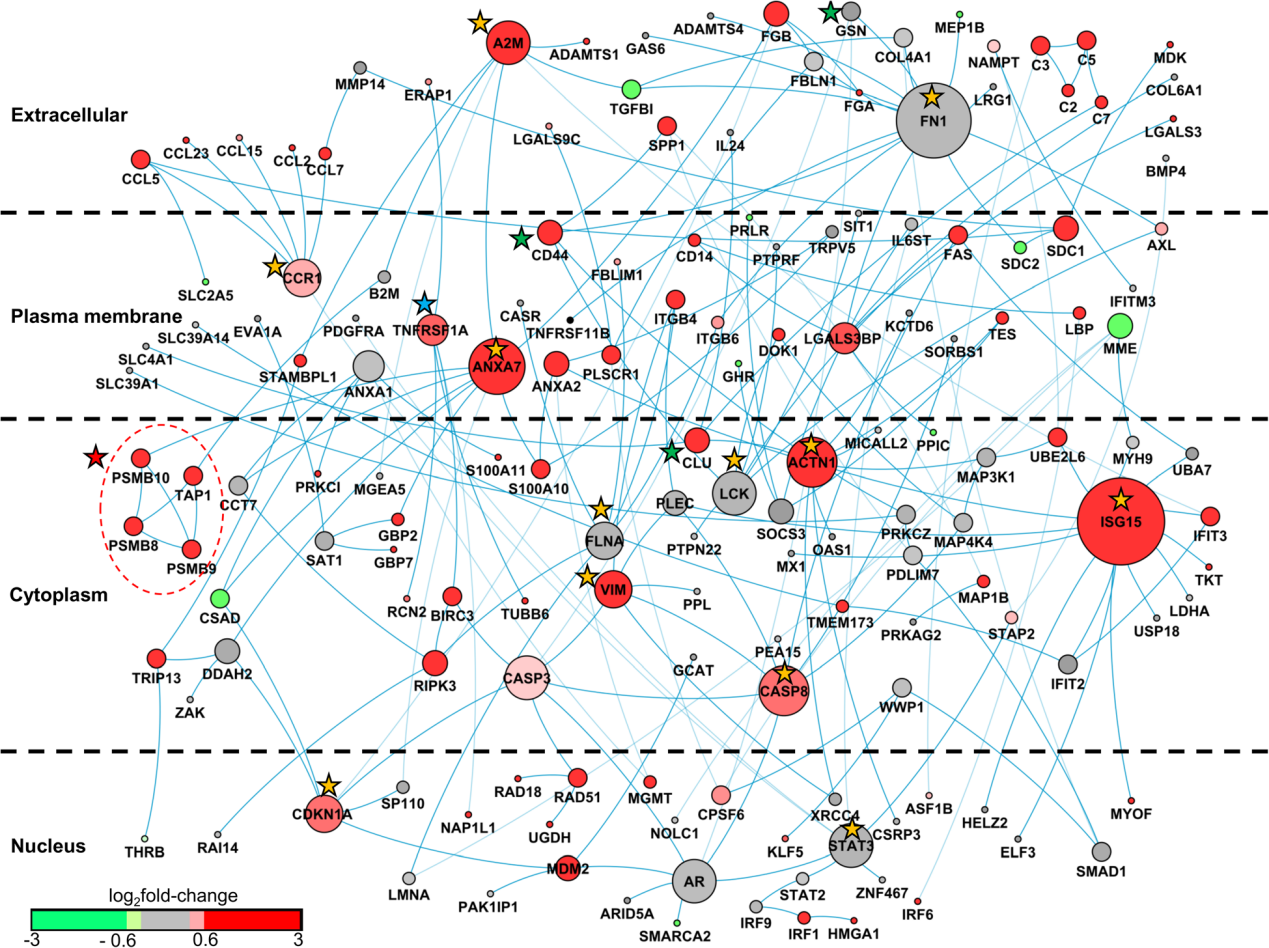
Acute kidney injury (AKI)-relevant human protein-protein interaction sub-network. The protein nodes are distributed according to cellular localization. The size of the node represents the number of connections in the sub-network. The nodes are colored according to the average log_2_ fold-change ratio in chemical exposures that cause kidney necrosis. Proteins encoded by genes with average log_2_ fold-change ratios greater than 0.6 are shown in *red*. Proteins encoded by genes with average log_2_ fold-change ratios between 0.6 and −0.6 are shown in *grey*. Proteins encoded by genes with average log_2_ fold-change ratios less than −0.6 are shown in *green. Orange stars* denote hub proteins with >5 connections, *green stars* denote non-hub proteins with a high *betweenness centrality* (>0.09). The *red star* and *dotted circle* identify the highest interconnected region of the network associated with the immunoproteasome

We carried out integrated analysis of gene expression data with PPI networks to generate disease-specific networks and gain new insights. We mapped the AKI-relevant gene set to the human high-confidence PPI network and extracted the connected component as a sub-network (AKI-SN) with 158 nodes and 196 edges, organized by cellular location (Fig. 6). We performed statistical significance tests based on random sampling and permutation to confirm that AKI-SN could not have been generated by random chance (Additional file 11: Figure S1). We calculated the statistical (or topological) properties of *degree* and *betweenness centrality* for all 158 proteins in AKI-SN (Additional file 12: Table S9), and ranked the proteins in the network according to these two properties (Additional file 13: Table S10). In network analysis, hub proteins represent proteins with a large *degree* (i.e., with the highest number of connections) and are hypothesized to play a key role in the network. *Betweenness centrality* (also known as *traffic*) represents the capacity of a protein node to facilitate interactions among members of the network, and is used to identify “communication hotspots” in the network [51]. The hub nodes with more than 5 connections were, in order of decreasing connectivity, protein products of the following genes: *Isg15*, *Fn1*, *Anxa7*, *Actn1*, *Casp8*, *Ar*, *A2m*, *Casp3*, *Stat3*, *Lck*, *Cdkn1a*, *Vim, Ccr1*, and *Flna* (see orange stars in Fig. 6). Many of these proteins are involved in AKI through a number of different biological processes, including the induction of apoptosis through *Casp8* and *Casp3* for nephrotoxic drugs [79], obstruction of tubular lumen by *Fn1* [80], and dysregulation of cytosolic calcium levels linked to *Anxa7* in connection with acute tubular necrosis [81–83]. The topmost hub node (*n* = 14 connections in the AKI-SN) was *Isg15* (interferon-stimulated gene 15), associated with apoptosis [84] and nephrotoxicant exposure in fish orthologs [85, 86]. Combined with this limited evidence in the literature, our network analysis predicts that *Isg15* may be a key AKI-related gene. We ranked the AKI-SN proteins according to *betweenness centrality* and identified non-hub nodes such as *Clu*, *Cd44*, and *Gsn* (see the green stars in Fig. 6). The protein products of *Clu* and *Gsn* (gelsolin) are well-documented biomarkers of AKI [80, 87]. The protein product of *Cd44* is a cell-surface glycoprotein receptor and acts as an adhesion molecule; *Cd44* and its ligand *Spp1* (osteopontin) are upregulated after ischemic kidney injury [88]. Our network analysis identified this node as a critical component of the AKI-SN despite the limited number of connections. In the AKI-SN, we captured the interaction between *Cd44* and *Spp1* (both upregulated). In addition to hub and high-traffic nodes, other AKI-associated genes mapped to AKI-SN (e.g., *Lgals3*, *Mdm2*, *S100A11*, *S100A10*, *SDC1,* and *A2M* [80, 89, 90] and the chemokine genes *Ccl2*, *Ccl5*, and *Ccl23*) [91]).

Next, we identified the highly interconnected regions in AKI-SN (Additional file 14: Figure S2). The most interconnected regions in disease-specific networks have been shown to capture disease-associated candidate genes [92]. The most interconnected region in AKI-SN contained the protein products of genes *Psmb8* (LMP7), *Psmb9* (LMP2), *Psmb10* (MECL1), and *Tap1*, which are involved in antigen processing and presentation (see the red star and circle in Fig. 6). The proteins of *Psmb8*, *Psmb9*, and *Psmb10* form the immunoproteasome—an alternative form of the normal constitutive proteasome—which is induced by interferon (IFN)-γ and TNF-α [93]. Immunoproteasomes are more efficient than constitutive proteasomes at eliminating damaged cellular proteins under severe stress or other pathological conditions, and are considered as promising drug targets in certain cancers and auto-immune diseases [93, 94]. Interferon regulatory factor-1 (IRF1) is a transcription factor that regulates the IFN-γ-mediated upregulation of immunoproteasome sub-units [95]. The protein product of the gene *Irf1* mapped to the AKI-SN (located in the nucleus region of Fig. 6), and was upregulated together with the immunoproteasome sub-units present in the network. As immunoproteasomes are promising drug targets, we further evaluated the expression of immunoproteasome sub-units across all 220 chemical exposures. We found that immunoproteasomes were upregulated by confirmed nephrotoxicants (e.g., lead-II-acetate, lead-IV-acetate, netilmicin, cisplatin, vancomycin, cholecalciferol, neomycin, gentamicin, and 2-amino-nitro-phenol; Additional file 15: Table S11). Analysis of chemical exposures associated with downregulation of immunoproteasome gene expression showed an interesting class effect: Anticancer drugs such as methotrexate as well as anthracycline anticancer agents, such as doxorubicin, daunorubicin, and epirubicin, were associated with diminished expression of genes related to immunoproteasomes.

The AKI-SN also contained regulatory elements of immunoproteasomes related to the TLR and TNF signaling pathways. The *Irf*-class of transcription factors are master regulators of TLRs and RIG-1 signaling pathways [96]. Genes of this class (e.g., *Irf1*, *Irf6*, and *Irf9*) mapped to the AKI-SN network in the nucleus. Components SPP1, LBP, and CCL5 of the TLR signaling pathway [97], located in the extracellular region and plasma membrane, were also upregulated. The presence of the TNF signaling pathway [67] in our network revealed a sequential connection between TNFRSF1A in the plasma membrane (marked with a blue star in Fig. 6) and BIRC3, RIPK3, and CASP3 in the cytosol. The bulk of these components showed strong upregulation, indicative of a strong and persistent immune and inflammatory response to the chemical insults. Our results are concordant with earlier studies that have documented potential immunoproteasome involvement in patients with IgA nephropathy [98] and patients rejecting renal transplants [99].

### Anatomical location

**Fig. 7:**
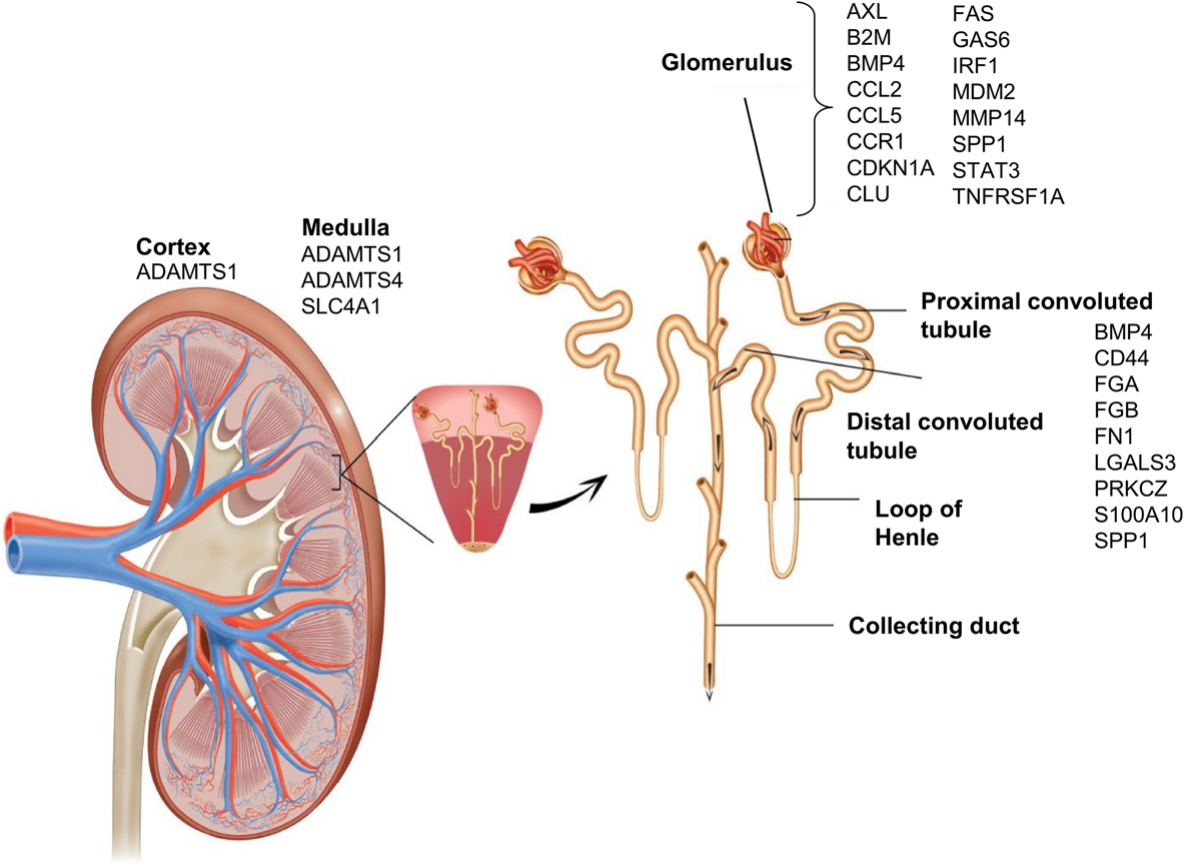
Acute kidney injury-subnetwork genes associated with different anatomical regions of the kidney

The Predictive Safety Testing Nephrotoxicity Working Group recently published criteria for a renal safety biomarker. One of the key characteristics is the ability of the biomarker to accurately localize damage to discrete anatomical units within the kidney architecture [10]. A literature review determined which potential biomarkers in our AKI-SN of 158 genes have been mapped to specific anatomical locations in the kidney (Fig. 7; Additional file 16: Table S12). The genes *Spp1*, *Lgals3*, *Fn1*, *CD44*, and *Bmp4* have been associated with the proximal tubular region of the kidney by microarray analysis and mouse gene knockout studies with experimentally induced acute kidney injury [100, 101]. Genes associated with glomerular injury included *Ccl2*, *Cdkn1a*, *Clu*, *Mmp14*, *Spp1*, and *Tnfrsf1a*. [102–107] (see Additional file 16: Table S12 for complete results of the IPA gene-to-function literature analysis).

### Frequently co-expressed genes with *Havcr1*

**Fig. 8:**
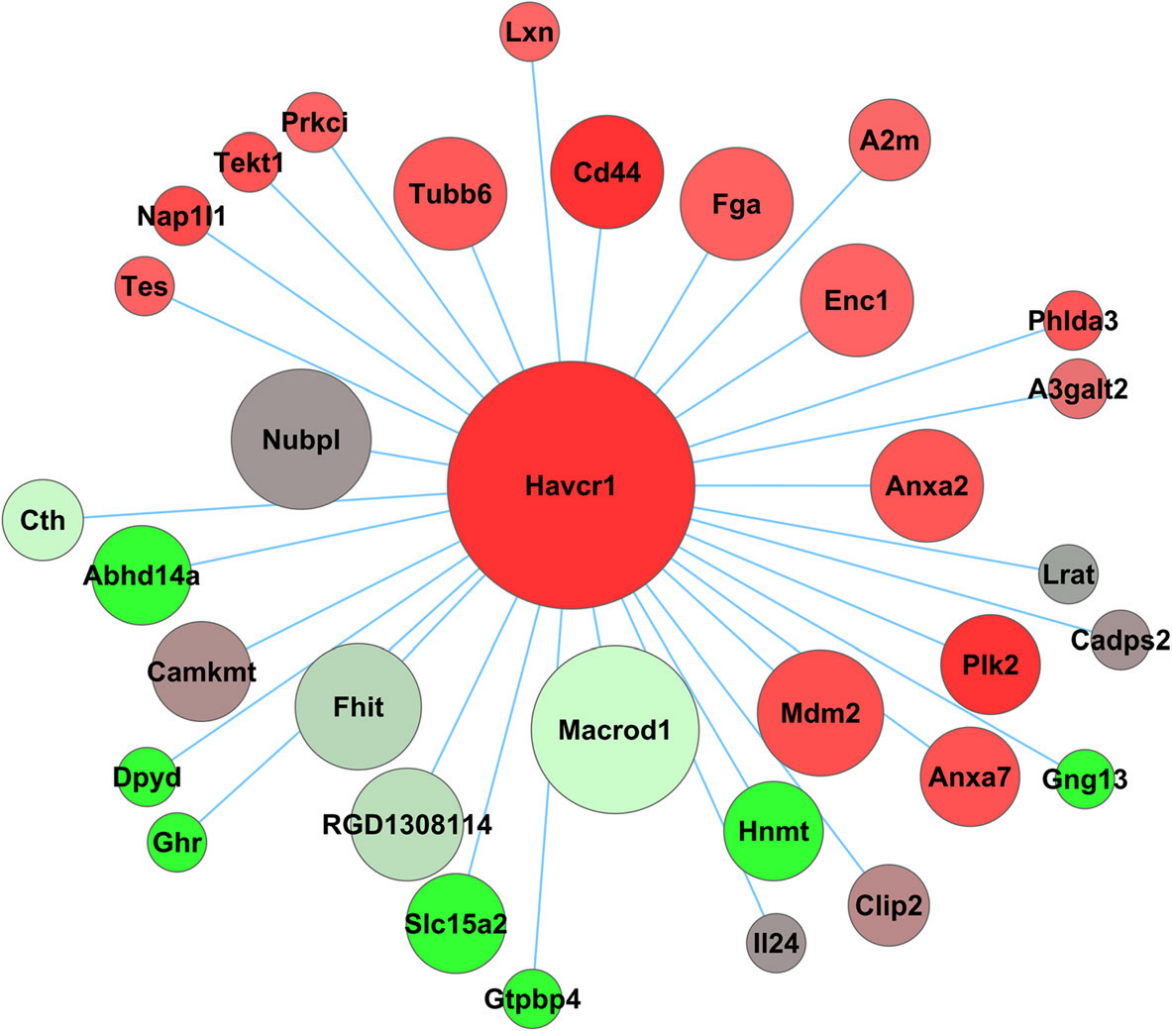
Frequently co-expressed genes with *Havcr1*. The size of the node represents the number of times the gene was co-expressed with *Havcr1* in the modules. The nodes are colored according to the average log_2_ fold-change ratio in chemical exposures causing kidney necrosis. Proteins encoded by genes with average log_2_ fold-change ratios greater than 0.6 are shown in *red*. Proteins encoded by genes with average log_2_ fold-change ratios between 0.6 and −0.6 are shown in grey. Proteins encoded by genes with average log_2_ fold-change ratios less than −0.6 are shown in *green*

We used our module-based analysis to identify network constituents of the FDA-qualified pre-clinical biomarker of AKI, *Havcr1* (commonly known as kidney injury molecule-1 or KIM-1). Modules were defined by using a variety of chemical exposures, and genes that co-expressed with one particular query gene under diverse chemical exposures helped to identify new genes that participate in the same pathway or biological process. Thus, we identified 33 genes that frequently co-expressed with *Havcr1*, i.e., the “*Havcr1*-co-expression gene set” (Fig. 8). Genes including *Cd44*, *Anxa2*, *Anxa7*, and *Mdm2*, which have been documented in AKI [101], were upregulated. We first ascertained that the *Havcr1*-co-expression gene set could not be identified from shuffled, randomized data (see Methods). We further analyzed the robustness of the gene set by leaving out either 5 % or 10 % of the chemical exposures and rerunning the analysis. In the leave-out 5 % analysis, *Il24* dropped out of the gene set and *A3galt* was identified the least number of times. In the leave-out 10 % analysis, *Cadps2* dropped out of the gene set, and *Il24* and *A3galt* were identified most infrequently. Overall, our analysis showed that the set of 33 genes that frequently co-expressed with *Havcr1* was significantly different from that expected by chance and robust with respect to its composition. Although most genes were unaffected, genes such as *Il24*, *A3galt*, and *Cadps2* could be de-prioritized. Our literature review confirmed that many genes in the *Havcr1*-co-expression gene set are associated with kidney injury. Of particular note was the potential for cross-regulation between *Cd44* and *Havcr1. Cd44* is not detectable in normal kidneys, but is expressed in the proximal tubule after acute ischemic injury [64]. *Cd44* is a cell adhesion molecule involved in biological processes similar to *Havcr1*, including the ability to phagocytose apoptotic cells [108], and its downregulation by *NF-κB* through the PI3K pathway. With *Cd44, Macrod1* in the network is also an essential gene for NF-*κB* activation [109]. *Cd44* mediates both phagocytosis of apoptotic cells [110] and anti-apoptotic signaling through the PI3K pathway [111], including PKC activation and influx of extracellular calcium after proteolytic cleavage of its ectodomain [112]. The evidence for possible cross-regulation between *Cd44* and *Havcr1,* including analysis of cleaved ectodomain fragments of *Cd44* as urinary marker of AKI, is a potentially testable hypothesis in future studies.

**Fig. 9:**
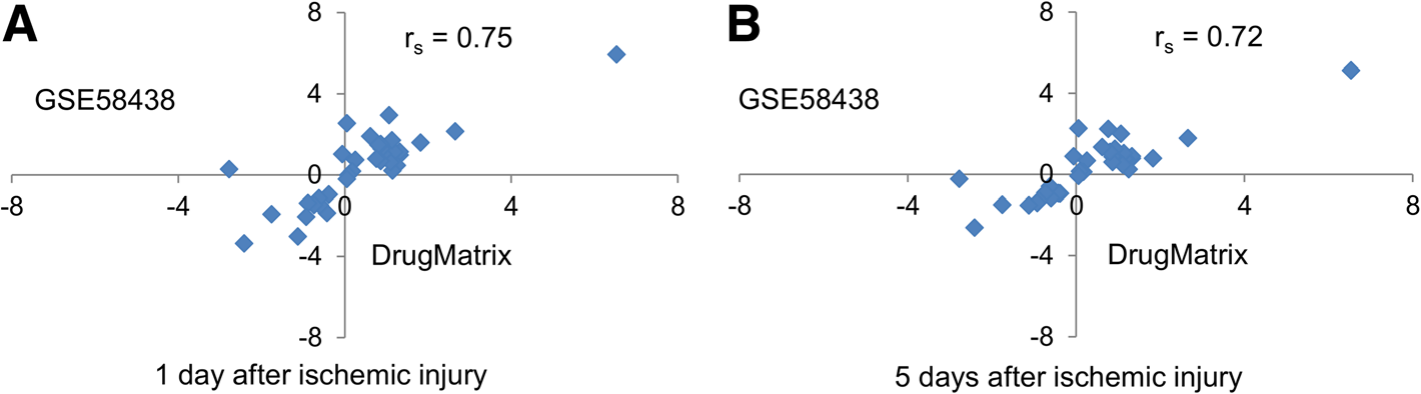
Scatterplots of log_2_ fold-change ratio for the genes in the *Havcr1*-co-expression gene set from the DrugMatrix data (x-axis) and an external rat kidney ischemic injury data set (GSE58438) (y-axis) at **a**) 1 day and **b**) 5 days after ischemic injury. The gene set is highly correlated in both data sets at both 1 and 5 days after ischemic injury, indicating a similar response to both chemically and non-chemically induced kidney injuries

We used an external rat kidney ischemic injury dataset from GEO to compare expression levels for the *Havcr1*-co-expression gene set across data sets. The external rat kidney ischemic injury dataset correlated strongly with the Drug-Matrix fold-changes, showing Spearman correlation coefficients (*r*_*s*_) of 0.75 and 0.72 at 1 and 5 days after injury, respectively (Fig. 9).

## Conclusions

Publicly available toxicogenomic datasets are valuable resources that help in data mining and identifying networks, mechanisms, and biomarkers associated with a disease. In this analysis, we studied the rat kidney toxicogenomic data from DrugMatrix and identified co-expression modules associated with kidney injury. Our work provides a compendium of kidney co-expression modules and is the first such analysis of kidney toxicogenomic data. We used the co-expression modules to identify a 30-gene signature that predicted the future onset of kidney injury from early gene transcription data. Although some of the genes in our signature are associated with the mechanism of AKI, we also identified genes such as *Irf6* as potentially novel candidates for AKI. Systems-level analyses identified pathways and networks associated with AKI. We identified AKI-relevant pathways, such as ECM receptor interaction and the RIG-1 signaling pathway, and showed that co-expression module-based approaches can identify additional information not obtainable by standard differential gene expression analysis. We identified an AKI-relevant protein interaction sub-network that mapped many known genes involved in AKI. Our network analysis revealed the involvement of immunoproteasomes in AKI and identified new genes, such as *Isg15* and *Anxa7*, not previously associated with this disease. Finally, we used our co-expression modules to identify the frequently co-expressed genes with known biomarker *Havcr1*. Overall, our analyses show the potential utility of using co-expression modules in characterizing molecular mechanisms involved in AKI and identifying novel mechanism-based biomarker candidates.

## Additional files

Additional file 1: Script S1. R script used to generate modules by using the iterative signature algorithm. (ZIP 15173 kb)
Additional file 2: Table S1. List of chemical exposures associated with different phenotypes and chemical exposure classes. (DOCX 19 kb)
Additional file 3: Script S2. R script used to convert Affymetrix probe ids to human gene IDs. (ZIP 3 kb)
Additional file 4: Table S2. List of genes associated with 137 co-expression modules. (XLSX 1398 kb)
Additional file 5: Table S3. List of chemical exposures associated with 137 co-expression modules. (XLSX 80 kb)
Additional file 6: Table S4. List of the AKI-relevant genes. (XLSX 74 kb)
Additional file 7: Table S5. List of chemical exposures that map to modules in module cluster 7 and module cluster 11. (XLSX 16 kb)
Additional file 8: Table S6. List of the 30 genes present in the AKI gene signature. (DOCX 15 kb)
Additional file 9: Table S7. Performance of 30-gene signature with external datasets. (DOCX 15 kb)
Additional file 10: Table S8. KEGG pathway enrichment analysis for differentially expressed genes. (DOCX 16 kb)
Additional file 11: Figure S1. Statistical analysis of AKI-relevant sub-network (AKI-SN). (DOCX 174 kb)
Additional file 12: Table S9. List of nodes in 158 AKI-relevant sub networks and their associated topological properties. (XLSX 19 kb)
Additional file 13: Table S10. List of highest ranking nodes when prioritized according to degree and betweenness centrality. (DOCX 17 kb)
Additional file 14: Figure S2. List of highly connected regions in AKI-relevant sub-network (AKI-SN). (DOCX 228 kb)
Additional file 15: Table S11. List of chemical exposures modulating the expression of immunoproteasomes in kidney. (XLSX 15 kb)
Additional file 16: Table S12. Complete list of publications supporting gene-to-function relationship in the 158 AKI-relevant sub-network genes (AKI-SN). (XLSX 17 kb)
