# Supplementary material for: Mining kidney toxicogenomic data by using gene co-expression modules
Source: BMC Genomics. 2016 Oct 10;17:790. doi: 10.1186/s12864-016-3143-y (PMC5057266; doi:10.1186/s12864-016-3143-y)
Supplement: Additional file 2: Table S1. — List of chemical exposures associated with different phenotypes and chemical exposure classes. (DOCX 19 kb) [file 12864_2016_3143_MOESM2_ESM.docx]

**Additional files**

**Mining kidney toxicogenomics data using gene co-expression modules**

Mohamed Diwan M. AbdulHameed,^1^ Danielle L. Ippolito,^2^ Jonathan D. Stallings,^2^ and Anders Wallqvist^1^

^1^Department of Defense Biotechnology High Performance Computing Software Applications Institute, Telemedicine and Advanced Technology Research Center, U.S. Army Medical Research and Materiel Command, Fort Detrick, Maryland 21702, USA

^2^U.S. Army Center for Environmental Health Research, 568 Doughten Drive, Fort Detrick, MD 21702, USA

**Additional File 2**

**Table S1. Chemical exposures associated with different injury phenotypes and chemical classes**

| Chemical | Dose (mg/kg) | Duration (days) | Phenotype |
| --- | --- | --- | --- |
| 2-Amino-4-nitrophenol | 625 | 5 | P1 |
| Lead-II-acetate | 600 | 3 | P1 |
| Lead-IV-acetate | 600 | 5 | P1 |
| 2-Amino-4-nitrophenol | 625 | 5 | P2 |
| Lead-II-acetate | 600 | 3 | P2 |
| Calcitriol | 0.04 | 5 | P2 |
| Cholecalciferol | 8 | 5 | P2 |
| Furosemide | 375 | 5 | P2 |
| Nimesulide | 162 | 5 | P2 |
| Vancomycin | 160 | 5 | P2 |
| 1-Naphthylisothiocyanate | 60 | 3 | C1 |
| 1-Naphthylisothiocyanate | 60 | 7 | C1 |
| Enrofloxacin | 2000 | 3 | C2 |
| Gatifloxacin | 770 | 5 | C2 |
| Norfloxacin | 1500 | 5 | C2 |
| Temafloxacin | 1000 | 3 | C2 |
| Temafloxacin | 1000 | 5 | C2 |
| Erlotinib | 116 | 3 | C3 |
| Erlotinib | 116 | 5 | C3 |
| Gefitinib | 116 | 3 | C3 |
| Gefitinib | 116 | 5 | C3 |
| β-Estradiol | 150 | 3 | C4 |
| β-Estradiol | 150 | 5 | C4 |
| Atrovastatin | 300 | 3 | C5 |
| Cerivastatin | 7 | 3 | C5 |
| Cerivastatin | 7 | 5 | C5 |
| Fluvastatin | 94 | 3 | C5 |
| Fluvastatin | 94 | 5 | C5 |
| Lovastatin | 1500 | 3 | C5 |
| Lovastatin | 1500 | 5 | C5 |
| Bezafibrate | 100 | 3 | C6 |
| Bezafibrate | 100 | 5 | C6 |
| Clofibrate | 500 | 3 | C6 |
| Fenofibrate | 215 | 3 | C6 |
| Fenofibrate | 215 | 5 | C6 |
| Gemfibrozil | 700 | 7 | C6 |
