# Supplementary material for: Mining kidney toxicogenomic data by using gene co-expression modules
Source: BMC Genomics. 2016 Oct 10;17:790. doi: 10.1186/s12864-016-3143-y (PMC5057266; doi:10.1186/s12864-016-3143-y)
Supplement: Additional file 8: Table S6. — List of the 30 genes present in the AKI gene signature. (DOCX 15 kb) [file 12864_2016_3143_MOESM8_ESM.docx]

**Additional files**

**Mining kidney toxicogenomics data using gene co-expression modules**

Mohamed Diwan M. AbdulHameed,^1^ Danielle L. Ippolito,^2^ Jonathan D. Stallings,^2^ and Anders Wallqvist^1^

^1^Department of Defense Biotechnology High Performance Computing Software Applications Institute, Telemedicine and Advanced Technology Research Center, U.S. Army Medical Research and Materiel Command, Fort Detrick, Maryland 21702, USA

^2^U.S. Army Center for Environmental Health Research, 568 Doughten Drive, Fort Detrick, MD 21702, USA

**Additional File 8**

**Table S6. List of genes present in the 30-gene signature**

| Gene symbol |
| --- |
| *Actn1*  *Amacr*  *Asf1b*  *B4galt6*  *Dhx58*  *Dnase1*  *Emx1*  *Flna*  *Fmo4*  *Guca2a*  *Havcr1*  *Igtp*  *Irf6*  *LOC100910973*  *Ly96*  *Map4k4*  *Mob3a*  *Mturn*  *Olfml2b*  *Plcl1*  *PVR*  *Rad51*  *Sapcd2*  *Scg5*  *Sorcs1*  *Tcrb*  *Tkt*  *Trip13*  *Ugt2b7*  *Uhrf1* |
