# Supplementary material for: Mining kidney toxicogenomic data by using gene co-expression modules
Source: BMC Genomics. 2016 Oct 10;17:790. doi: 10.1186/s12864-016-3143-y (PMC5057266; doi:10.1186/s12864-016-3143-y)
Supplement: Additional file 9: Table S7. — Performance of 30-gene signature with external datasets. (DOCX 15 kb) [file 12864_2016_3143_MOESM9_ESM.docx]

**Additional files**

**Mining kidney toxicogenomics data using gene co-expression modules**

Mohamed Diwan M. AbdulHameed,^1^ Danielle L. Ippolito,^2^ Jonathan D. Stallings,^2^ and Anders Wallqvist^1^

^1^Department of Defense Biotechnology High Performance Computing Software Applications Institute, Telemedicine and Advanced Technology Research Center, U.S. Army Medical Research and Materiel Command, Fort Detrick, Maryland 21702, USA

^2^U.S. Army Center for Environmental Health Research, 568 Doughten Drive, Fort Detrick, MD 21702, USA

**Additional File 9**

**Table S7. Performance of 30-gene signature with external datasets**

| Dataset | Sensitivity | Specificity | Accuracy |
| --- | --- | --- | --- |
| TG-GATEs^a^ - 3days | 83% | 75% | 79% |
| TG-GATEs^a^ - 7days | 80% | 56% | 68% |

^a^TG-GATEs - Toxicogenomics Project-Genomics Assisted Toxicity Evaluation System
