# Supplementary material for: Mining kidney toxicogenomic data by using gene co-expression modules
Source: BMC Genomics. 2016 Oct 10;17:790. doi: 10.1186/s12864-016-3143-y (PMC5057266; doi:10.1186/s12864-016-3143-y)
Supplement: Additional file 10: Table S8. — KEGG pathway enrichment analysis for differentially expressed genes. (DOCX 16 kb) [file 12864_2016_3143_MOESM10_ESM.docx]

**Additional files**

**Mining kidney toxicogenomics data using gene co-expression modules**

Mohamed Diwan M. AbdulHameed,^1^ Danielle L. Ippolito,^2^ Jonathan D. Stallings,^2^ and Anders Wallqvist^1^

^1^Department of Defense Biotechnology High Performance Computing Software Applications Institute, Telemedicine and Advanced Technology Research Center, U.S. Army Medical Research and Materiel Command, Fort Detrick, Maryland 21702, USA

^2^U.S. Army Center for Environmental Health Research, 568 Doughten Drive, Fort Detrick, MD 21702, USA

**Additional File 10**

**Table S8. KEGG pathway enrichment for differentially expressed genes^a^**

| Pathway | Count | p-value^b^ |
| --- | --- | --- |
| TNF^c^ signaling pathway | 12 | 1.10^-5^ |
| Complement and coagulation cascades | 10 | 3.10^-5^ |
| Chemical carcinogenesis | 9 | 4.10^-4^ |
| Glutathione metabolism | 7 | 5.10^-4^ |
| Renin-angiotensin system | 6 | 1.10^-3^ |

^a^ We calculated the differentially expressed genes (DEG) for each of the chemical exposures that produce kidney injury phenotypes (P1 and P2 in **Table S1**) and selected those that were differentially expressed in at least two of those chemical exposures. We used the rank product method and considered all genes with a False Discovery Rate equal to or less than 0.05 to be differentially expressed genes. We identified 360 DEGs and performed the KEGG pathway enrichment analysis as described earlier in the method section.

^b^ Benjamini-Hochberg multi-test corrected p-value, ^c^ Tumor necrosis factor
