# Supplementary material for: Mining kidney toxicogenomic data by using gene co-expression modules
Source: BMC Genomics. 2016 Oct 10;17:790. doi: 10.1186/s12864-016-3143-y (PMC5057266; doi:10.1186/s12864-016-3143-y)
Supplement: Additional file 13: Table S10. — List of highest ranking nodes when prioritized according to degree and betweenness centrality. (DOCX 17 kb) [file 12864_2016_3143_MOESM13_ESM.docx]

**Additional files**

**Mining kidney toxicogenomics data using gene co-expression modules**

Mohamed Diwan M. AbdulHameed,^1^ Danielle L. Ippolito,^2^ Jonathan D. Stallings,^2^ and Anders Wallqvist^1^

^1^Department of Defense Biotechnology High Performance Computing Software Applications Institute, Telemedicine and Advanced Technology Research Center, U.S. Army Medical Research and Materiel Command, Fort Detrick, Maryland 21702, USA

^2^U.S. Army Center for Environmental Health Research, 568 Doughten Drive, Fort Detrick, MD 21702, USA

**Additional File 13**

**Table S10. Topological properties of top-prioritized proteins in the acute kidney injury (AKI)-relevant sub-network**

| Protein | Degree | Betweenness centrality |
| --- | --- | --- |
| ISG15 | 14 | 0.22 |
| FN1 | 12 | 0.29 |
| ANXA7 | 9 | 0.14 |
| ACTN1 | 8 | 0.16 |
| CASP8 | 7 | 0.14 |
| AR | 7 | 0.21 |
| CASP3 | 7 | 0.21 |
| STAT3 | 7 | 0.19 |
| A2M | 7 | 0.14 |
| LCK | 7 | 0.09 |
| CDKN1A | 6 | 0.11 |
| VIM | 6 | 0.11 |
| CCR1 | 6 | 0.07 |
| FLNA | 6 | 0.06 |
| LGALS3BP | 5 | 0.07 |
| TNFRSF1A | 5 | 0.05 |
| ANXA1 | 5 | 0.03 |
| CD44 | 4 | 0.10 |
| CLU | 4 | 0.09 |
| GSN | 3 | 0.12 |
