# Supplementary material for: Mining kidney toxicogenomic data by using gene co-expression modules
Source: BMC Genomics. 2016 Oct 10;17:790. doi: 10.1186/s12864-016-3143-y (PMC5057266; doi:10.1186/s12864-016-3143-y)
Supplement: Additional file 14: Figure S2. — List of highly connected regions in AKI-relevant sub-network (AKI-SN). (DOCX 228 kb) [file 12864_2016_3143_MOESM14_ESM.docx]

**Additional files**

**Mining kidney toxicogenomics data using gene co-expression modules**

Mohamed Diwan M. AbdulHameed,^1^ Danielle L. Ippolito,^2^ Jonathan D. Stallings,^2^ and Anders Wallqvist^1^

^1^Department of Defense Biotechnology High Performance Computing Software Applications Institute, Telemedicine and Advanced Technology Research Center, U.S. Army Medical Research and Materiel Command, Fort Detrick, Maryland 21702, USA

^2^U.S. Army Center for Environmental Health Research, 568 Doughten Drive, Fort Detrick, MD 21702, USA

**Additional File 14**


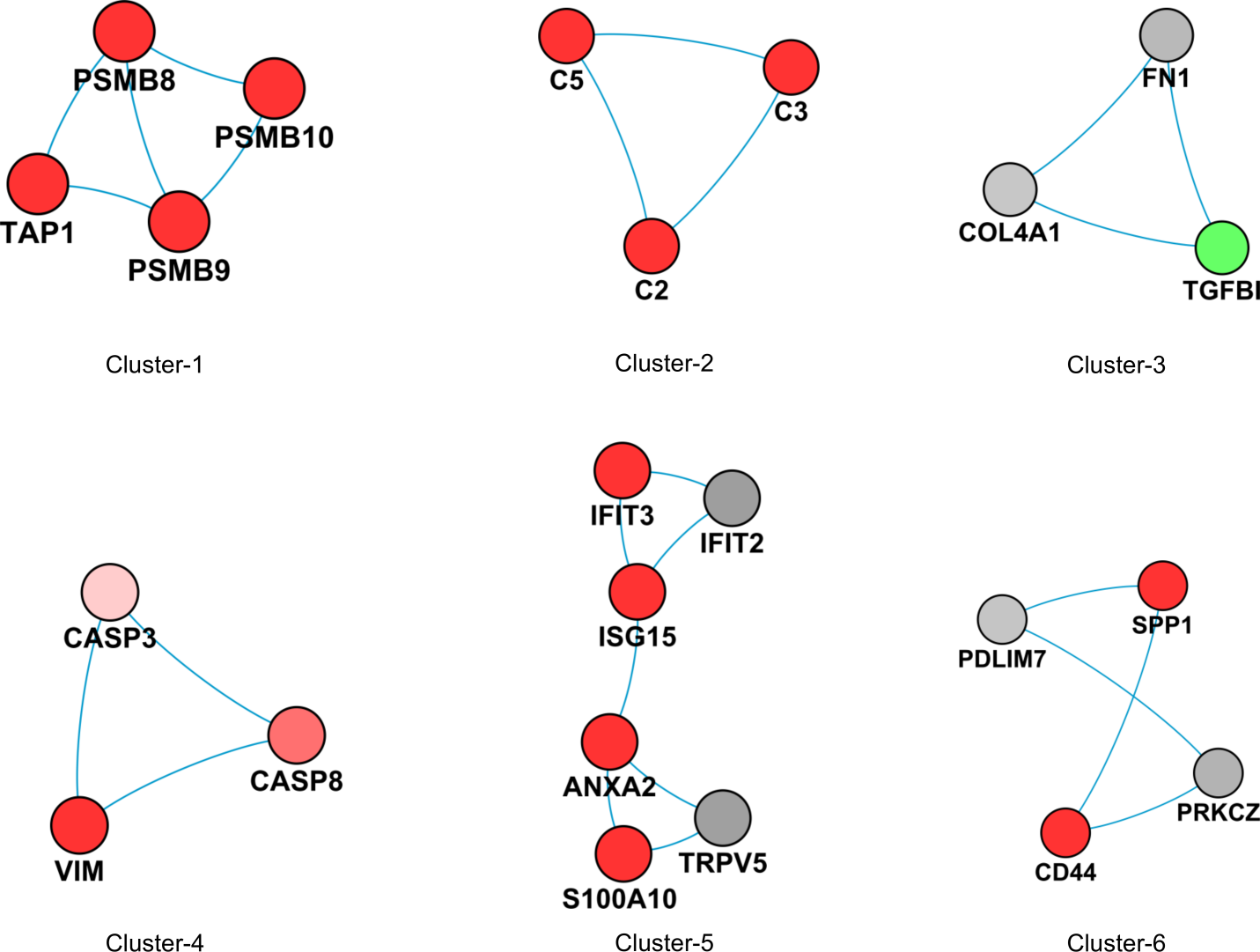


**Figure S2.** List of highly interconnected regions in acute kidney injury (AKI)-relevant sub-network (AKI-SN).
